# Supplementary material for: Causal Relationship between Aging and Anorexia Nervosa: A White-Matter-Microstructure-Mediated Mendelian Randomization Analysis
Source: Biomedicines. 2024 Aug 16;12(8):1874. doi: 10.3390/biomedicines12081874 (PMC11351342; doi:10.3390/biomedicines12081874)
Supplement: Supplementary file 1 [file biomedicines-12-01874-s001.zip › Supplementary Figure/Figure S4/Singe SNP plot of MR analysis for TL to ubm-b-1873.pdf]

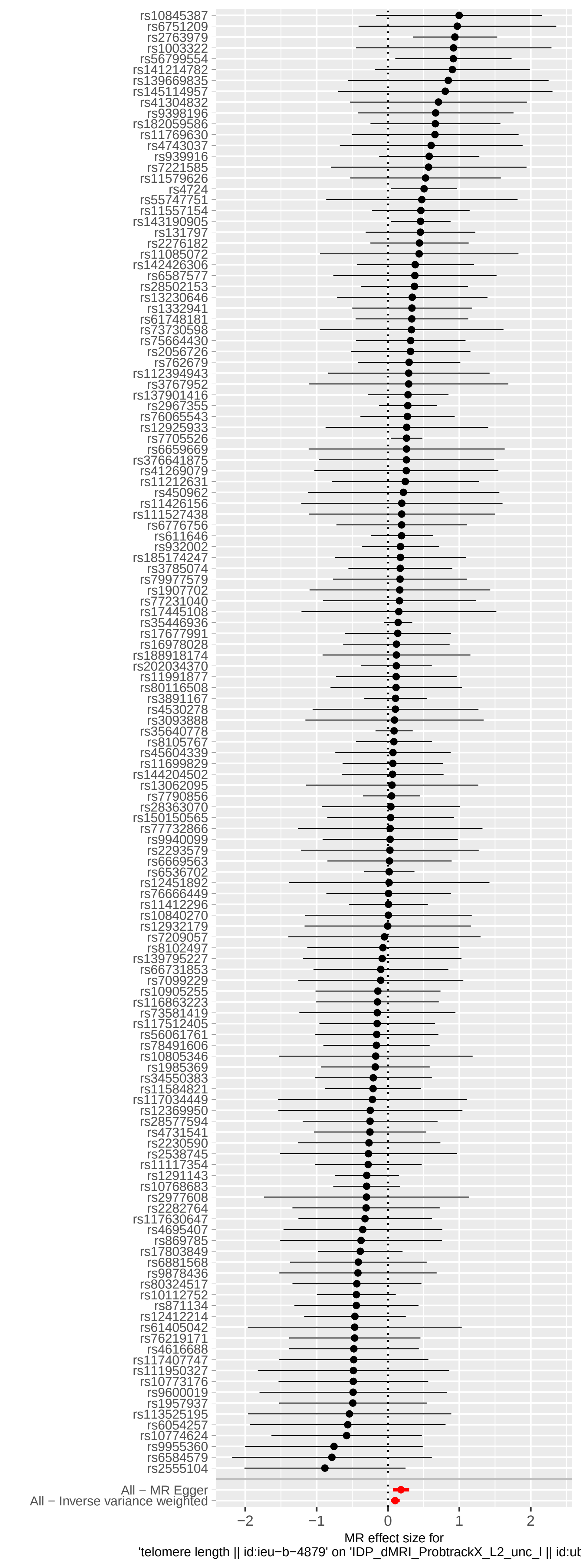
rs10845387
rs6751209
rs2763979
rs1003322
rs56799554
rs141214782
rs139669835
rs145114957
rs41304832
rs9398196
rs182059586
rs11769630
rs4743037
rs939916
rs7221585
rs11579626
rs4724
rs55747751
rs11557154
rs143190905
rs131797
rs2276182
rs11085072
rs142426306
rs6587577
rs28502153
rs13230646
rs1332941
rs61748181
rs73730598
rs75664430
rs2056726
rs762679
rs112394943
rs3767952
rs137901416
rs2967355
rs76065543
rs12925933
rs7705526
rs6659669
rs376641875
rs41269079
rs11212631
rs450962
rs11426156
rs111527438
rs6776756
rs611646
rs932002
rs185174247
rs3785074
rs79977579
rs1907702
rs77231040
rs17445108
rs35446936
rs17677991
rs16978028
rs188918174
rs202034370
rs11991877
rs80116508
rs3891167
rs4530278
rs3093888
rs35640778
rs8105767
rs45604339
rs11699829
rs144204502
rs13062095
rs7790856
rs28363070
rs150150565
rs77732866
rs9940099
rs2293579
rs6669563
rs6536702
rs12451892
rs76666449
rs11412296
rs10840270
rs12932179
rs7209057
rs8102497
rs139795227
rs66731853
rs7099229
rs10905255
rs116863223
rs73581419
rs117512405
rs56061761
rs78491606
rs10805346
rs1985369
rs34550383
rs11584821
rs117034449
rs12369950
rs28577594
rs4731541
rs2230590
rs2538745
rs11117354
rs1291143
rs10768683
rs2977608
rs2282764
rs117630647
rs4695407
rs869785
rs17803849
rs6881568
rs9878436
rs80324517
rs10112752
rs871134
rs12412214
rs61405042
rs76219171
rs4616688
rs117407747
rs111950327
rs10773176
rs9600019
rs1957937
rs113525195
rs6054257
rs10774624
rs9955360
rs6584579
rs2555104
All − MR Egger
All − Inverse variance weighted
−2
−1
0
1
2
MR effect size for
'telomere length || id:ieu−b−4879' on 'IDP_dMRI_ProbtrackX_L2_unc_l || id:ub
